# Socio-Economic Inequity: Diabetes in New Zealand

**DOI:** 10.3389/fmed.2022.756223

**Published:** 2022-05-10

**Authors:** Lui Holder-Pearson, James Geoffrey Chase

**Affiliations:** Department of Mechanical Engineering, Centre for Bioengineering, University of Canterbury, Christchurch, New Zealand

**Keywords:** diabetes, inequity, rationing of care, hardware commoditization, unaffordable care, low-cost alternatives, insulin pump (CSII)

## 1. Introduction

New Zealand is on collision course with the iceberg of unaffordable medicine. Diabetes is one example which has seen — and is projected to see — significant growth, stressing the health system. A decreasing workforce supporting an aging population, combined with a considerable increase in the prevalence, means gold-standard care will be unobtainable except for an increasingly select few, with public resources distributed among hundreds of thousands of sufferers. There are currently an estimated 228,000 New Zealanders suffering from type-2 diabetes alone, with a projected growth of 70–90%, to approximately 400,000 people by 2,040 (1). Considering rates are higher and outcomes worse among Māori and Pasifika (1, 2), and they typically face greater socio-economic hardship (3), there is a double burden. The current healthcare system seeks intrinsic advancements—increased productivity without increased efficiency. Demographic and prevalence challenges beg for extrinsic changes: innovative disruptions to current care models, combating growing inequity of access to care and outcomes.

## 2. Diabetes—Is It Really *That* Bad? the Human Cost

Diabetes seems to have become so common, even those suffering from it claim to be of good health (4). Active management involves medication to: (a) artificially increase the sensitivity to insulin; (b) stimulate further production of insulin; and finally (c) supplement with external insulin. The aim is to lower circulating blood glucose concentrations. High blood glucose results in harm to many body structures, causing significant complications, cumulative cost, life-limiting disability (5, 6), and early death (1, 7–10).

The human cost of diabetes is paid for disproportionately by certain ethnic and socio-economic groups. In New Zealand, Māori are 2.5× more likely to have type-2 diabetes than their Pkeh counterparts, with prevalence of 7.5% compared to the national average of 4.7%. Pasifka peoples are further over-represented, with an estimated prevalence of 15.1% (1). Even given the same access to primary healthcare, there is an inequity of outcome (11), with Māori and Pasifika having a HbA1C 11–13 mmol/mol higher, after adjusting for both medical management and demographic factors (2, 12). Adjusted for ethnicity, lower household income alone is correlated with a 2× risk of diabetes mellitus (13). Trends in outcomes are also significantly worse, with the most deprived now 3× more likely to die from cardiovascular pathology than the least deprived, compared to equal risk two decades prior (11).

## 3. Is It Really *That* Bad? the Financial Cost

While overall health spending has increased from 7.5% of GDP to 9.2% since 2000 (14), publicly-funded treatment diabetes and its complications now costs New Zealand 0.67% of GDP, and ≈10% of the total health budget, or $2.1B NZD per annum (1). Predictions estimate by 2,040, diabetes will cost New Zealand $3.5 billion in 2021 dollars, equal to 16% of the current health budget.

Systemic health costs from diabetes are growing, but personal health costs are rising faster to cover the gap. Private spending accounts for approximately 20% of total health expenditure in New Zealand (15), of which 12–15% is directly an out-of-pocket expense (14). Added to explicit expenses, is considerable personal loss from lost wages due to activity-impairing complications, and lost non-salary productivity due to the inability to perform activities such as domestic cares or voluntary work (1). Lost personal wages have an estimated economic cost of $562 m in 2020, but increase 47%–$755 m in 2,040, and non-salary economic loss is predicted to increase from $334 to $506 m in the same period (1). The lost wages are accompanied by a loss of government revenue through income tax, from $163 m in 2020 to $221 m in 2,040. Lost tax revenue because of disability from diabetes is equal to 8% of the current governmental health expenditure on diabetes (1).

The large projected growth of financial costs of treating diabetes is driven by increasing prevalence, population growth, aging population, and higher costs per patient because of earlier diagnoses. In particular, the average lifetime cost is 13 times greater when diagnosed with type 2 diabetes at 25-years, compared to 75-years [$565 vs. $44 k (1)]. Previous screening trials indicated almost one in five people had pre-diabetes in 2008/2009. These trends, especially among Māori and Pasifika mean the healthcare system cannot afford to delay either actively acting to prevent type 2 diabetes, or introduce extrinsic changes to provide equitable access to more effective management.

## 4. The Only Positive Thing About Inequity Is the Feedback Loop

**Figure 1:**
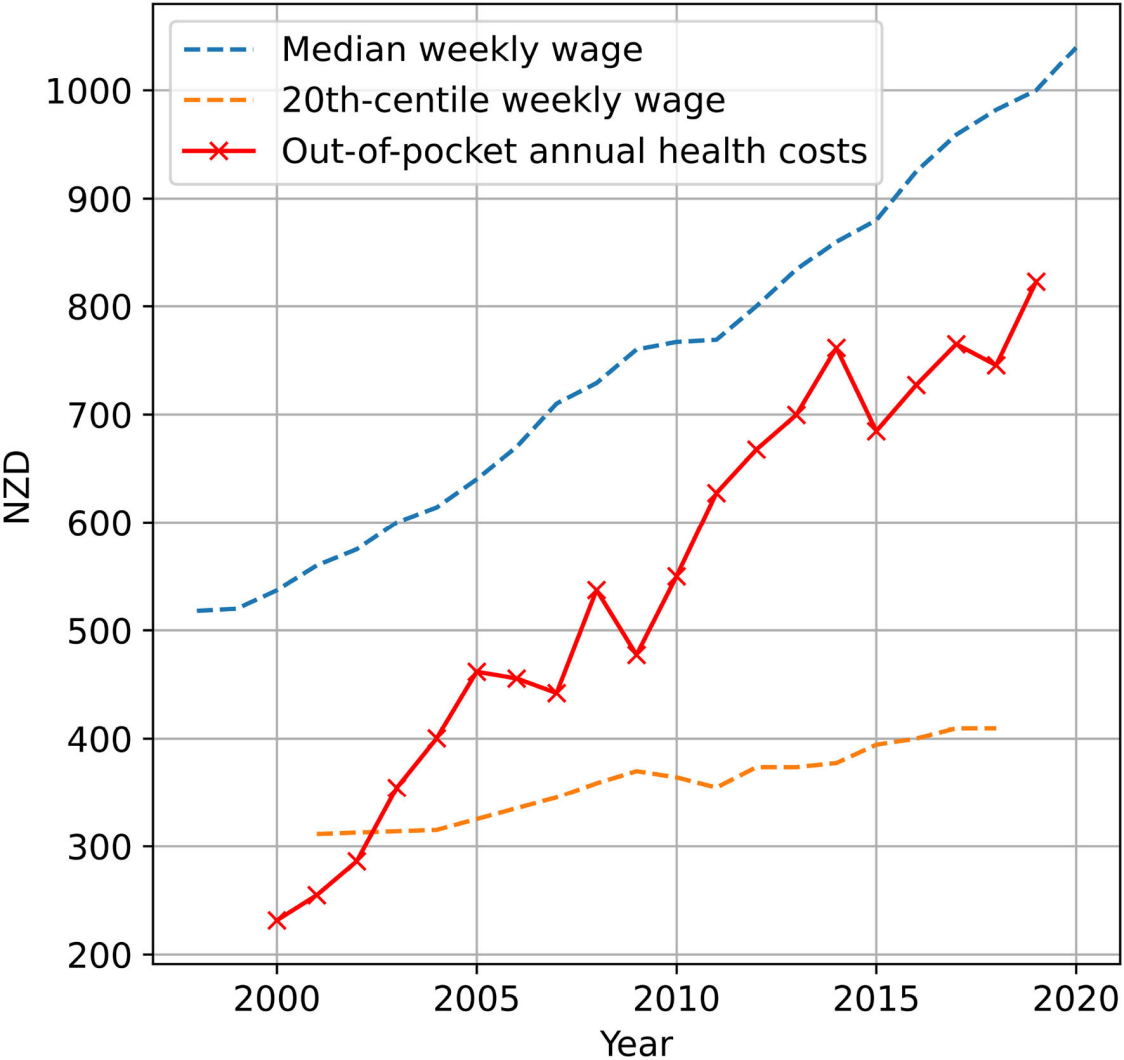
Historic average annual out-of-pocket health expenditure per capita (14), compared to median weekly wage (16, 17), and mean of 20th-centile weekly household income (16). Out-of-pocket health expenses are exclusive of charitable and private insurance spending. Average historic annual increases are 4.3% for out-of-pocket healthcare expenditure, compared to 3.2% for median wage growth, and only 1.5% for the 20th centile.

Diabetes inequities are not only worsening, but self-perpetuating. Financially, inequity is increasing due to increases in out-of pocket spending, as shown in Figure 1. Accounting for private insurances and charitable spending, the most recent data from the Ministry of Health show out-of-pocket expenditure increased on average 4.3% per annum (albeit in 2012 when it was last reported). This rate compares to an average inflation rate of 2.7% (18), and an average median wage growth of 3.2% (3). To exaggerate inequity further, the mean household income of the 20th centile [P20 from Table 9.1 of (16)] has risen on average only 1.5% per annum, a cohort which Māori are 30% more likely to be in than European descendants (3). These financial impediments to healthcare are seen in the ability to access primary healthcare, something 38% of Māori report as being unable to do (19).

Forming a disproportionately high amount of the lower socio-economic cohort (3), Māori have worse diabetes outcomes compared to their pkeh counterparts (2, 20). One possible explanation is less equitable access to primary care (2), as evidenced in lower-income households having 25% fewer GP appointments relative to the portion who report being of ‘poor health’, than NZ average (21). Even accounting for government subsidy schemes, such as the community services card, a GP appointment costs up to $19.50. The average household in the lowest quintile in New Zealand has only $230 per week disposable income after housing (3). **Visiting a general practitioner for the most vulnerable is as much, if not more, a financial decision as a healthcare one**.

Projections for out-of-pocket diabetes-related spending are expected to increase 3.0% per annum, rising almost 80% in the two decades to 2,040 (1), similar to historic median wage growth, but two times the 20th centile. As primary healthcare is expected to see the largest rises in out-of-pocket health expenditure in GP appointments and prescription co-payments (1), access to preventative healthcare becomes less equitable, impeding early intervention and compounding inequity of outcomes for healthcare. This inequity is compounded by the positive feedback loop of poor health literacy (22, 23), which furthers distrust in the profession as a whole.

Inequity of access to both care and outcomes in diabetes is a positive-feedback loop of increasing loss of equity, with resultant harm and cost, which requires extrinsic, innovative changes to current care models to break.

## 5. One Small Driver for Big Change

One technology which provides better control for individuals with both type-one and type-two diabetes (24, 25), but is available only to a minority of New Zealanders, is insulin pumps. Pumps in New Zealand are up to $10,000 if self-funded, or, if stringent requirements are met, publicly funded (26). Criteria for public funding are evidence the pump will reduce HbA1C by 10 mmol/mol, or “*four severe unexplained hypoglycaemic episodes*" requiring medical assistance (27). If not publicly-funded, there are additional out-of-pocket costs for consumables approximately $1,500 per year. In poorer households, these required out-of-pocket costs, compounded by poorer health literacy, are preventing equitable access to best care (28, 29).

An open-licence pump developed at the University of Canterbury in partnership with local diabetes clinicians, is currently being prototyped with a bill of materials approximately $\frac{1}{50}$ the cost of the devices currently available—the ultra-low cost insulin pump (ULCIP) with comparable performance (30). Accounting for comfortable production, support, and development overheads would enable purchase of the device at $500 per device - enabling 14-20× the number of insulin pumps for no added investment, which would allow significant relaxation of the insulin pump eligibility criteria, thus increasing the equity of access to best care.

The flow-on benefits of widespread access to insulin pumps are such extrinsic changes required to address growing health demands. Currently, secondary care spending accounts for 57% of diabetes spending, a portion which is expected to grow (1). Widespread adoption of insulin pumps for no additional investment would see an increase in good glucose control, which subsequently reduces complication rate and severity (2, 24, 31). This reduces the number, and cost, of secondary care presentations for individuals with diabetes, allowing for further investment in preventative medicine, increasing equitable access to better health outcomes.

## 6. Discussion

The inequities present in healthcare in New Zealand, specifically diabetes, are worsening in light of increasing prevalence and severity of diabetes. Increases in the personal out-of-pockets costs of healthcare are the result of explicit rationing of care: an implicit redefinition of the social contract, where systemic healthcare is becoming less accessible and less thorough in its application. Māori, who have a history of underinvestment and poor engagement, continue to suffer disproportionately in a public health system considered “*hostile and alienating*" (32). Within diabetes specifically, inequities in outcomes have been recognized for over two decades (2). Despite this recognition, there has been a lack of progress to address outcomes through increased access or similar schemes. This lack of progress is evident in the lack of access to specialist care (2), and thus technologies that require prescription by a specialist (33).

These results generalize broadly to other indigenous populations, where disparities of care still exist around diabetes and care in general (34–36). Equally, some European focused studies show similar results around socio-economic status (37). Hence, there are international studies showing similar trends as the New Zealand studies showing growing inequity, where this analysis provides a first overall analysis of how these outcomes translate into inequity in access to care and outcomes.

Despite the financial trends highlighted, representative of “*persisting inequities ... in access and outcomes for Māori, Pacific Island, and low-income populations*” (19), comparisons between out-of-pocket expenditure and wages are lacking. This analysis links the lack of access to a fundamental cause on the part of the patient. There is academic agreement at a system level, inequities remain in the health system as a continuation of inequities generated through colonization (38), and reinforced by continued poverty, lack of access to housing, and lack of access to primary healthcare (19, 38). However, the economic and more specific reasons have not been to date explicitly analyzed.

More specifically, the reasons for the trends observed include continued underinvestment of the public health system, resulting in a larger portion of healthcare expenditure shifted to private expenditure (19). Considering the New Zealand healthcare funding policies where secondary care is fully publicly funded, but primary healthcare is not, primary healthcare has become increasingly unaffordable for those with the greatest socio-economic deprivation. This gap is particularly evident where only the wealthier 28.8% of individuals possess private healthcare insurance (19), allowing them to obtain private secondary care ahead of rationing in a system under stress.

The results of these are evident in the significantly higher rates of emergency department re-admission (39) for Māori, suggesting that where follow-up care from a primary healthcare provider may be appropriate, Māori are instead disproportionately seeking it through emergency departments. Missing primary care due to economic and availability reasons poses inequitable access to preventative care, thus patients presenting to emergency departments are also more complex, exacerbating potentially avoidable negative outcomes. Hence, there is a strong, economically-driven feedback loop where patients unable to access primary care suffer negative outcomes, and for Māori in particular, these reasons are both socio-economic as well as ethnic in origin.

Healthcare inequities due to increased rationing of care require solutions which enable increased productivity without an increase in resources. A broadly interoperable insulin pump paired with an equally openly-accessible glucose monitor (40) would enable a low-cost, equitable artificial pancreas system (LEAPS). In addition to improved patient-led diabetes care, a LEAPS solution would also more timely and efficient clinician assistance through cloud-based processing for patient-level and system-wide analysis.

## Author Contributions

LH-P developed and researched the exact extent of the problems and led writing. JC provided considerable insight, context, and editing. All authors contributed to the article and approved the submitted version.

## Funding

This work was supported by the NZ National Science Challenge 7, Science for Technology and Innovation (2019-S3-CRS).

## Conflict of Interest

The authors declare that the research was conducted in the absence of any commercial or financial relationships that could be construed as a potential conflict of interest.

## Publisher's Note

All claims expressed in this article are solely those of the authors and do not necessarily represent those of their affiliated organizations, or those of the publisher, the editors and the reviewers. Any product that may be evaluated in this article, or claim that may be made by its manufacturer, is not guaranteed or endorsed by the publisher.

## References

[1] Shepard-Wipiiti T Brennan L. The Economic and Social Cost of Type 2 Diabetes [resreport]. Ministry of Health (2021). Available online at: https://healthierlives.co.nz/report-on-the-economic-and-social-cost-of-type-2-diabetes/.

[2] Jansen RM Sundborn G Cutfield R Yu D Simmons D. Ethnic inequity in diabetes outcomes-inaction in the face of need. N Z Med J. (2020) 133:8–10. Available online at: https://journal.nzma.org.nz/journal-articles/ethnic-inequity-in-diabetes-outcomes-inaction-in-the-face-of-need 33223543

[3] Statistics New Zealand Tatauranga Aotearoa. Household Income and Housing-Cost Statistics: Year ended June 2020. (2021). Available online at: https://www.stats.govt.nz/information-releases.

[4] Dalbo VJ Teramoto M Roberts MD Scanlan AT. Lack of reality: positive self-perceptions of health in the presence of disease. Sports. (2017) 5:23. 10.3390/sports5020023 29910383 PMC5968989

[5] Oseni-Momodu E Chima A Lengman S. Pains of amputation amongst diabetic foot ulcer patients in north central Nigeria: amputation versus no amputation. Nigerian J Family Pract. (2018) 9:68–75.

[6] Xue M Xu W Ou YN Cao XP Tan MS Tan L. Diabetes mellitus and risks of cognitive impairment and dementia: a systematic review and meta-analysis of 144 prospective studies. Ageing Res Rev. (2019) 55:100944. 10.1016/j.arr.2019.100944 31430566

[7] Norhammar A Mellbin L Cosentino F. Diabetes: Prevalence, prognosis and management of a potent cardiovascular risk factor. Eur J Prev Cardiol. (2017) 24:52–60. 10.1177/2047487317709554 28618910

[8] Wright AK Kontopantelis E Emsley R Buchan I Sattar N Rutter MK. Life expectancy and cause-specific mortality in type 2 diabetes: a population-based cohort study quantifying relationships in ethnic subgroups. Diabetes Care. (2017) 40:338–45. 10.2337/dc16-1616 27998911

[9] Zhou M Liu J Hao Y Liu J Huo Y Smith SC. Prevalence and in-hospital outcomes of diabetes among patients with acute coronary syndrome in China: findings from the Improving Care for Cardiovascular Disease in China-Acute Coronary Syndrome Project. Cardiovasc Diabetol. (2018) 17:1–14. 10.1186/s12933-018-0793-x 30482187 PMC6258152

[10] Walker J Colhoun H Livingstone S McCrimmon R Petrie J Sattar N. Type 2 diabetes, socioeconomic status and life expectancy in Scotland (2012-2014): a population-based observational study. Diabetologia. (2018) 61:108–16. 10.1007/s00125-017-4478-x 29075822 PMC6448945

[11] Yu D Zhao Z Osuagwu UL Pickering K Baker J Cutfield R. Ethnic differences in mortality and hospital admission rates between Māori, Pacific, and European New Zealanders with type 2 diabetes between 1994 and 2018: a retrospective, population-based, longitudinal cohort study. The Lancet Global Health. (2021) 9:e209–17. 10.1016/S2214-109X(20)30412-5 33069275

[12] Simmons D Kenealy T Scott DJ. Implementing the South Auckland diabetes plan: barriers and lessons. N Z Med J. (2000) 113:364–6. Available online at: https://www.proquest.com/docview/1138546217 11130372

[13] Metcalf PA Scragg RR Schaaf D Dyall L Black PN Jackson RT. Comparison of different markers of socioeconomic status with cardiovascular disease and diabetes risk factors in the Diabetes, Heart and Health Survey. N Z Med J. (2008) 121:45–56. 18278081

[14] The World Bank. Indicators. (2021). Available online at: https://data.worldbank.org/indicator/SH.XPD.CHEX.GD.ZS?locations=NZ.

[15] Ministry of Health. Health Expenditure Trends in New Zealand 2000–2010. Wellington: Ministry of Health (2012).

[16] Perry B. Household Incomes in New Zealand: Trends in Indicators or Inequality and Hardship 1982 to 2018. Wellington: Ministry of Social Development (2019).

[17] Statistics New Zealand Tatauranga Aotearoa. NZ.Stat. (2021). Available online at: http://nzdotstat.stats.govt.nz/wbos/Index.aspx.

[18] Reserve Bank. Inflation Calculator. (2021). Available online at: https://www.rbnz.govt.nz/monetary-policy/inflation-calculator.

[19] Goodyear-Smith F Ashton T. New Zealand health system: universalism struggles with persisting inequities. Lancet. (2019) 394:432–42. 10.1016/S0140-6736(19)31238-3 31379334

[20] Kenealy T Elley C Robinson E Bramley D Drury P Kerse N. An association between ethnicity and cardiovascular outcomes for people with Type 2 diabetes in New Zealand. Diabet Med. (2008) 25:1302–8. 10.1111/j.1464-5491.2008.02593.x 19046220

[21] Thomson M. Who had access to doctors before and after new universal capitated subsidies in New Zealand? Health Policy. (2019) 123:756–64. 10.1016/j.healthpol.2019.04.004 31213333

[22] Stormacq C Van den Broucke S Wosinski J. Does health literacy mediate the relationship between socioeconomic status and health disparities? Integrative review. Health Promot Int. (2019) 34:e1e17. 10.1093/heapro/day062 30107564

[23] Schmidt-Busby J Wiles J Exeter D Kenealy T. Understandings of disease among Pacific peoples with diabetes and end-stage renal disease in New Zealand. Health Expect. (2019) 22:1122–31. 10.1111/hex.12946 31368649 PMC6803558

[24] Morera J Joubert M Morello R Rod A Lireux B Reznik Y. Sustained efficacy of insulin pump therapy in type 2 diabetes: 9-year follow-up in a cohort of 161 patients. Diabetes Care. (2016) 39:e74e75. 10.2337/dc16-0287 27208322

[25] Landau Z Raz I Wainstein J Bar-Dayan Y Cahn A. The role of insulin pump therapy for type 2 diabetes mellitus. Diabetes Metab Res Rev. (2017) 33:e2822. 10.1002/dmrr.2822 27189155

[26] Pharmac. Insulin Pumps and Consumables. (2020). Available online at: https://pharmac.govt.nz/medicine-funding-and-supply/.

[27] Pharmac. Online Pharmaceutical Schedule. - August 2021. (2021). Available online at: https://schedule.pharmac.govt.nz/ScheduleOnline.php?osq=Insulin%20pump&code=C0115123968.

[28] McKergow E Parkin L Barson DJ Sharples KJ Wheeler BJ. Demographic and regional disparities in insulin pump utilization in a setting of universal funding: a New Zealand nationwide study. Acta Diabetol. (2017) 54:63–71. 10.1007/s00592-016-0912-7 27650535

[29] Sa'uLilo L Tautolo ES Egli V Smith M. Health literacy of Pacific mothers in New Zealand is associated with sociodemographic factors and non-communicable disease risk factors: surveys, focus group and interviews. J Pacific Res. (2018) 21:67–70. 10.26635/phd.2018.914

[30] Payne M Pooke F Chase JG Campbell J Holder-Pearson L Knopp J. The separation of insulin pump hardware and software - a novel and low-cost approach to insulin pump design. IFAC-PapersOnLine. (2021) 54:502–7. 10.1016/j.ifacol.2021.10.306

[31] Coppell KJ Drabble SJ Cochrane JA Stamm RA Sullivan TA. The cost of diabetes-related hospital care to the Southern District Health Board in 2016/17. N Z Med J. (2019) 132:35–45. 31647793

[32] Graham R Masters-Awatere B. Experiences of Māori of Aotearoa New Zealand's public health system: a systematic review of two decades of published qualitative research. Aust N Z J Public Health. (2020) 44:193–200. 10.1111/1753-6405.12971 32311187

[33] Hennessy LD Lange MD Wiltshire EJ Jefferies C Wheeler BJ. Youth and non-European ethnicity are associated with increased loss of publicly funded insulin pump access in New Zealand people with type 1 diabetes. Diabetic Medicine. (2020) 38:14450. 10.1111/dme.14450 33131079

[34] Bauchner H. Rationing of health care in the United States. JAMA. (2019) 321:751. 10.1001/jama.2019.1081 30758496

[35] Crowshoe L Dannenbaum D Green M Henderson R Hayward MN Toth E. Type 2 diabetes and indigenous peoples. Can J Diabetes. (2018) 42:S296306. 10.1016/j.jcjd.2017.10.022 29650108

[36] Maple-Brown LJ Graham S McKee J Wicklow B. Walking the path together: incorporating Indigenous knowledge in diabetes research. Lancet Diabet Endocrinol. (2020) 8:559–60. 10.1016/S2213-8587(20)30188-1 32559468

[37] Tatulashvili S Fagherazzi G Dow C Cohen R Fosse S Bihan H. Socioeconomic inequalities and type 2 diabetes complications: a systematic review. Diabet Metab. (2020) 46:89–99. 10.1016/j.diabet.2019.11.001 31759171

[38] Sharma S Walton M Manning S. Social determinants of health influencing the New Zealand COVID-19 response and recovery: a scoping review and causal loop diagram. Systems. (2021) 9:52. 10.3390/systems9030052

[39] Curtis E Paine SJ Jiang Y Jones P Tomash I Healey O. Examining emergency department inequities in Aotearoa New Zealand: Findings from a national retrospective observational study examining Indigenous emergency care outcomes. Emerg Med Austr. (2021) 34:16–23. 10.1111/1742-6723.13876 34651443 PMC9293399

[40] Campbell J Holder-Pearson L Pretty C Benton C Knopp J Chase J. Development of a NIR light absorption reflectance pulse glucometer. IFAC-PapersOnLine. (2020) 53:15970–5. 10.1016/j.ifacol.2020.12.388

